# The Comparison of mDCF and mFOLFOX-6 as First-Line Treatment in Metastatic Gastric Cancer

**DOI:** 10.7759/cureus.14882

**Published:** 2021-05-07

**Authors:** Yusuf Acikgoz, Selin Aktürk Esen, Gokhan Ucar, Merve Dirikoc, Yakup Ergun, Oznur Bal, Dogan Uncu

**Affiliations:** 1 Medical Oncology, Health of Science Ankara City Hospital, Ankara, TUR; 2 Medical Oncology, Batman City Hospital, Batman, TUR

**Keywords:** metastatic gastric cancer, mdcf, mfolfox-6, toxicity, survival rate

## Abstract

Introduction: Fluoropyrimidine and platinum-based chemotherapy regimens are widely accepted for metastatic gastric cancer (GC). Because of drug toxicity, a combined two-drug cytotoxic drug regimen is recommended for first-line therapy, while three-drug cytotoxic regimens are recommended for patients with medically fit and better performance status. In this study, it was aimed to compare modified FOLFOX-6 (mFOLFOX-6) and modified DCF (mDCF) regimens in terms of survival and side effects in first-line treatment in metastatic GC.

Methods: We retrospectively reviewed the clinical record of patients with metastatic gastric or gastro-esophageal junction cancer who had received mDCF or mFOLFOX-6 as the first-line treatment, and followed up in our center between February 2013 and December 2020. The data were collected from the patients' registration database of the hospital and oncologic follow-up files of our center. In the mDCF arm, docetaxel 60 mg/m^2 ^and cisplatin 60 mg/m^2^ on day 1 intravenous (i.v.) infusion, and 600 mg/m^2^ 5-fluorouracil (FU) as a continuous infusion for five days were administrated every three weeks for up to six cycles. In the mFOLFOX-6 arm, 85 mg/m^2 ^oxaliplatin and 400 mg/m^2^ LV as an i.v. infusion over two hours and a 5-FU bolus of 400 mg/m^2^ as a 10-minute infusion, followed by 2.400 mg/m^2^ 5-FU as a 46-hour continuous infusion were administrated every two weeks for up to six cycles. Univariate and multivariate analyses for overall survival (OS) were performed by Cox proportional hazards regression model. Survival analysis was performed by the Kaplan-Meier method with the Long-rank test. P-value <0.05 was considered statistically significant.

Results: A total of 70 patients included into the study. Of those, 40 (57%) patients had received mDCF and 30 (43%) had received FOLFOX-6 regimens as first-line treatment. There were no complete responses in both groups. The partial response rate was 28% and 27% for mDCF and mFOLFOX-6, respectively. There was no statistically significant difference regarding treatment response for both groups (p=0.787). The median OS was 13.9 months (95% CI: 7.5-20.4) in the mDCF arm, and 10.4 months (95% CI: 6.4-14.4) in the mFOLFOX-6 arm (p=0.409). The median progression-free survival (PFS) was 5.2 months (95% CI: 3.6-6.9) in the mDCF arm, and 6.4 months (3.2-9.6) in the FOLFOX-6 arm (p=0.126). The ratio of dose reduction, treatment delay, and neutropenic fever were not statistically different between treatment arms.

Conclusion: The present study demonstrated that proper patient selection for metastatic GC may give rise to comparable survival rates without increased toxicity. mFOLFOX-6 and mDCF had similar response rates, OS, PFS, and side effect profiles.

## Introduction

Although the incidence of gastric cancer (GC) has decreased in the USA and Western European countries in recent years, it still continues to be an important problem in Central Asian countries [1-5]. GC is the fifth most common cancer in the world and is the third in cancer-related deaths [6]. The incidence of GC may differ 15-20 times according to geographical regions [1]. While GC is the most common type of cancer in men in Japan and Korea, it is the fifteenth most common cancer-related death in the USA.

Fluoropyrimidine and platinum-based chemotherapy regimens are widely accepted for metastatic GC therapy [7]. Because of drug toxicity, a combined two-drug cytotoxic drug regimen is recommended for first-line therapy, while three-drug cytotoxic regimens are recommended for patients who are medically fit and with better performance status. Trastuzumab can be added to first-line chemotherapy with fluoropyrimidine and platinum-based agents in human epidermal growth factor receptor 2 (HER-2) positive metastatic gastric adenocarcinoma [7].

In this study, we aimed to compare modified FOLFOX-6 (mFOLFOX-6) and modified DCF (mDCF) regimens in terms of survival and side effects in first-line treatment in metastatic GC.

## Materials and methods

Patients

The data collected from the patients' registration database of the hospital and oncologic follow-up files were as follows: age at diagnosis, gender, ECOG performance score, presence of comorbid disease, baseline carcinoembryonic antigen (CEA) and CA 19-9 levels, tumor location, histological subtype, presence of liver or peritoneum metastasis, metastasis status (de novo vs recurrent), the number of metastasis, first-line treatment regimen, treatment response, treatment toxicity, and survival data.

Treatment arms

In the mDCF arm, docetaxel 60 mg/m^2^ and cisplatin 60 mg/m^2^ on day 1 intravenous (i.v.) infusion, and 600 mg/m^2^ 5-fluorouracil (FU) as a continuous infusion for five days were administrated every three weeks for up to six cycles.

In the mFOLFOX-6 arm, 85 mg/m^2^ oxaliplatin and 400 mg/m^2^ LV as an i.v. infusion over 2 hours and a 5-FU bolus of 400 mg/m^2^ as a 10-minutes infusion, followed by 2.400 mg/m^2^ 5-FU as a 46-hour continuous infusion were administrated every two weeks for up to six cycles.

Treatment response

Treatment response was evaluated according to RECIST 1.1 criteria for every 12 weeks with computerized tomography (CT). According to RECIST criteria, complete response (CR) included the disappearance of all target lesions and reduction in the short-axis measurement of all pathologic lymph nodes to ≤10 mm; partial response (PR) was defined as ≥30% decrease in the sum of the longest diameter of the target lesions compared with baseline; progressive disease (PD) was defined as ≥20% increase of at least 5 mm in the sum of the longest diameter of the target lesions compared with the smallest sum of the longest diameter recorded and the appearance of one or more new lesions; stable disease (SD) was considered for patients who met neither PR nor PD criteria.

Overall survival (OS) was defined as the time between the date of diagnosis of metastatic disease and the date of last control for alive patients or death from any cause. Progression-free survival (PFS) was defined as the time between the date of starting first-line treatment (mDCF or FOLFOX-6) and disease progression or death whichever occurred first.

Statistical analysis

Statistical analysis was performed by using Statistical Package for the Social Sciences Version 22.0 for Windows (IBM Corp., Armonk, NY, USA). The comparison of two groups were performed by Mann-Whitney U test and Pearson chi-square or Fisher's test for continuous and categorical variables, respectively. We used the Kaplan-Meier test for survival analysis, and outcomes were analyzed by the Log-rank test. Univariate and multivariate analyses for OS were performed by Cox proportional hazards regression model. All variables were included both into the univariate and multivariate analysis. We reported two-sided P-values, and P-value <0.05 considered statistically significant.

## Results

Patients characteristics

A total of 70 patients were included into the study. Of those, 40 (57%) patients had received mDCF and 30 (43%) had received FOLFOX-6 regimens as first-line treatment. The median age was 60 (22-76) years, and the number of male patients was 51 (73%). The number of patients who had a comorbid disease was 37 (53%) among all patients. The number of patients who had gastric tumor was 63 (90%), and 7 (10%) patients had gastro-esophageal junction tumor. The distribution of histological subtype was as follows: intestinal type was 49 (70%), the diffuse type was 17 (24%), and mixt type was 4 (6%). The number of patients who had de novo metastatic disease was 41 (59%), while 29 (41%) patients had recurrent disease.

The baseline clinical characteristics were not statistically different between treatment arms except for the presence of comorbid disease and metastasis status (Table 1). The ratio of patients who had a comorbid disease was higher in the FOLFOX arm compared to the mDCF arm (67% vs 42%, p=0.045). The number of patients who had de novo metastatic disease was higher in the mDCF arm compared to the mFOLFOX-6 arm (82% vs 27%, p<0.001).

**Table 1:** Baseline features of both groups and all patients. CEA: carcinoembryonic antigen, ECOG PS: Eastern Cooperative Oncology Group Performance Status. *p-value < 0.05 is statistically significant.

Characteristics	mDCF, n (%)	FOLFOX-6, n (%)	Total, n (%)	p-Value
Number of patients	40 (57)	30 (43)	70	
Median age (min-max)	55 (22–69)	62 (36–76)	60 (22–76)	0.082
Gender				0.244
Male	27 (67)	24 (80)	51 (73)	
Female	13 (33)	6 (20)	19 (27)	
Comorbid disease				0.045*
Yes	17 (42)	20 (67)	37 (53)	
No	23 (58)	10 (33)	33 (47)	
ECOG PS				0.680
0	18 (45)	13 (43)	31 (44)	
1	17 (43)	11 (37)	28 (40)	
2	5 (12)	6 (20)	11 (16)	
Baseline CEA level (ng/mL); median (min-max)	3.90 (0.77–950)	3.07 (0.50–1004)	3.50 (0.50–1004)	0.172
Baseline Ca 19–9 level (U/mL); median (min-max)	25 (0.80–1618)	9.2 (0.60–146)	9.9 (0.6–1618)	0.085
Tumor location				0.421
Gastric	35 (87)	28 (93)	63 (90)	
Gastro-esophageal junction	5 (13)	2 (7)	7 (10)	
Histological subtype				0.865
Intestinal	29 (73)	20 (67)	49 (70)	
Diffuse	9 (22)	8 (26)	17 (24)	
Mixt	2 (5)	2 (7)	4 (6)	
Metastasis status				<0.001*
De-novo metastasis	33 (82)	8 (27)	41 (59)	
Recurrent metastasis	7 (18)	22 (73)	29 (41)	
Liver metastasis				0.832
Yes	15 (38)	12 (40)	27 (39)	
No	25 (62)	18 (60)	43 (61)	
Peritoneum metastasis				0.622
Yes	17 (42)	11 (37)	28 (40)	
No	23 (58)	19 (63)	42 (60)	
The number of metastasis				0.676
Single metastasis	22 (55)	18 (60)	40 (57)	
Multiple metastases	18 (45)	12 (40)	30 (43)	
The duration of treatment; median cycle (min-max)	5 (3–6)	3.5 (2–6)	5 (2–6)	0.119
Dose reduction				0.928
Yes	20 (58)	15 (60)	35 (59)	
No	14 (42)	10 (40)	24 (41)	
Treatment delay				0.943
Yes	16 (47)	12 (48)	28 (48)	
No	18 (53)	13 (52)	31 (52)	
Neutropenic fever				0.635
Yes	7 (17)	4 (13)	11 (15)	
No	33 (83)	26 (87)	59 (85)	

Treatment response and toxicity

The median duration of treatment was 5 (3-6) cycles and 3.5 (2-6) cycles for mDCF and mFOLFOX-6, respectively. The rate of dose reduction, treatment delay, and neutropenic fever were not statistically different between treatment arms (Table 1). There was no complete response in both groups. The partial response rate was 28% and 27% for mDCF and mFOLFOX-6, respectively. There was no statistically significant difference regarding disease control rate (DCR) for both groups (0.787; Table 2).

**Table 2:** Treatment response for the first-line chemotherapy. CR: complete response, PR: partial response, SD: stable disease.

Characteristics	mDCF, n (%)	mFOLFOX-6, n (%)	p-Value
Disease control rate (CR+PR+SD)	24 (60)	20 (67)	0.568
Progressive disease	16 (40)	10 (33)	0.568

Survival analysis

**Figure 1:**
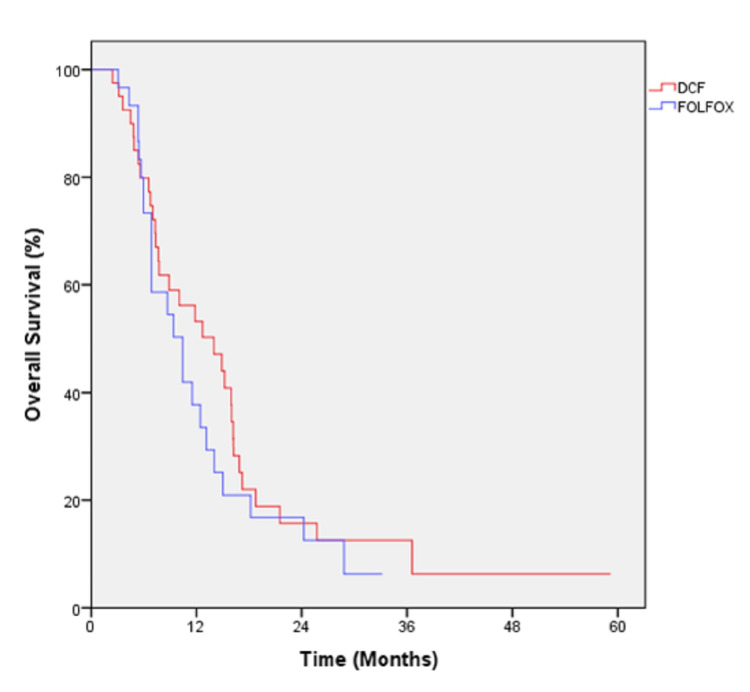
Overall survival of all patients by the first-line treatment.

**Figure 2:**
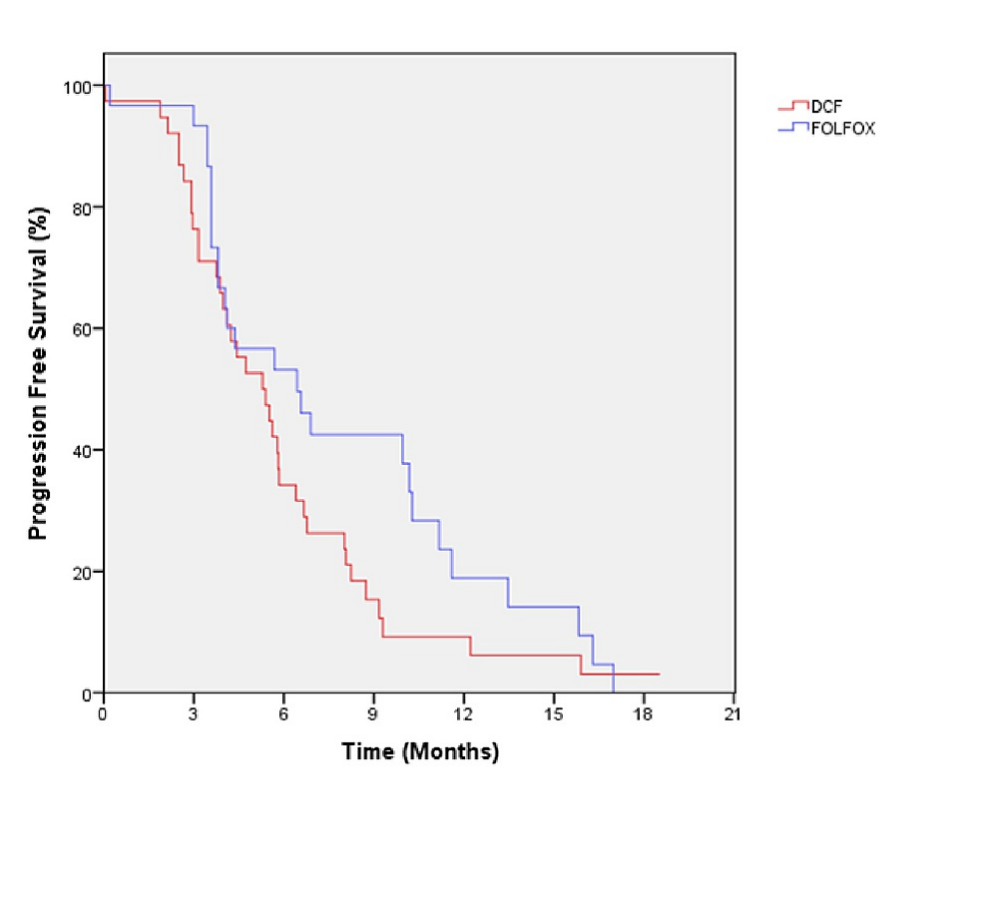
Progression-free survival of all patients by the first-line treatment.

The median follow-up was 10.5 months in the mDCF arm, and 7.8 months in the mFOLFOX-6 arm. The number of death was 56 (80%) in the entire cohort at the time of final analysis. The median OS was 13.9 months (95% CI: 7.5-20.4) in mDCF arm, and 10.4 months (95% CI: 6.4-14.4) in mFOLFOX-6 arm (p=0.409; Figure 1). The median PFS was 5.2 months (95% CI: 3.6-6.9) in the mDCF arm, and 6.4 months (3.2-9.6) in the FOLFOX-6 arm (p=0.126; Figure 2).

Univariate and multivariate analyses

Univariate and multivariate analyses were performed by defining OS as an endpoint. Age, gender, comorbid disease, ECOG PS, histological subtypes, treatment arms, metastasis status, liver metastasis, peritoneum metastasis, the number of metastasis were analyzed both in univariate and multivariate analysis. According to univariate analysis patients with peritoneum, metastasis had an increased risk for death compared to those without peritoneum metastasis with a hazard ratio (HR) of 1.92 (95% CI: 1.12-3.28; p=0.016). Whereas, there were no prognostic factors for OS according to multivariate analysis. The results of univariate and multivariate analysis are shown in Table 3.

**Table 3:** Univariate and multivariate analysis for OS. HR: hazard ratio, OS: overall survival, ECOG PS: Eastern Cooperative Oncology Group Performance Status. *p-value < 0.05 is statistically significant.

Variables (n)	Univariable	P-value	Multivariable	P-value
	HR (95% CI)		HR (95% CI)	
Age < 60 years > 60 years	10.77 (0.45–1.31)	0.349	10.94 (0.47–1.87)	0.866
Gender				
Male (51)	1	0.541	1	0.571
Female (19)	1.19 (0.66–2.15)		1.24 (0.58–2.62)	
Comorbid disease				
Yes (37)	1	0.682	1	0.885
No (33)	1.11 (0.65–1.90)		0.94 (0.45–1.98)	
ECOG PS				
0 (31)	1		1	
1 (28)	0.82 (0.46–1.46)	0.513	0.80 (0.42–1.54)	0.521
2 (11)	1.45 (0.67–3.13)	0.337	1.20 (0.49–2.95)	0.681
Histological subtype				
Intestinal (49)	1		1	
Diffuse (17)	1.66 (0.90–3.05)	0.099	2.00 (0.76–5.23)	0.154
Mixt (4)	0.91 (0.21–3.79)	0.898	0.96 (0.20–4.59)	0.962
Treatment arms				
DCF (40)	1	0.411	1	0.813
FOLFOX (30)	1.25 (0.73–2.14)		1.11 (0.46–2.67)	
Metastasis status				
De-novo metastasis (41)	1	0.495	1	0.333
Recurrent metastasis (29)	1.20 (0.70–2.05)		1.50 (0.65–3.45)	
Liver metastasis				
Yes (27)	1	0.907	1	0.266
No (43)	0.96 (0.56–1.65)		0.63 (0.28–1.41)	
Peritoneum metastasis				
No (42)	1	0.016*	1	0.104
Yes (28)	1.92 (1.12–3.28)		1.75 (0.89–3.45)	
The number of metastasis				
Single metastasis (40)	1	0.568	1	0.509
Multiple metastases (30)	1.16 (0.68–1.99)		1.27 (0.62–2.58)	

## Discussion

Various cytotoxic agents can be used in the metastatic GC: fluoropyrimidines (fluorouracil, capecitabine, and S-1), platins (cisplatin and oxaliplatin), taxanes (docetaxel and paclitaxel), anthracycline epirubicin, and irinotecan, which is a topoisomerase inhibitor [8]. Using these agents alone results in a low ORR. For example, the response is 20-40% with fluoropyrimidines [9-12], 20% with taxanes [13,14], and 20% with irinotecan [15]. Considering the toxicity, the addition of docetaxel to fluoropyrimidine and platinum-based chemotherapy in the first-line treatment is controversial [16]. Docetaxel has been used as a monotherapy and combination therapy in GC and its effectiveness has been demonstrated in several studies [17,18]. In a study, DCF (docetaxel 75 mg/m^2^ and cisplatin 75 mg/m^2^ on day 1 iv infusion, and 750 mg/m^2^ 5-FU as continuous infusion for five days every three weeks) and CF (cisplatin 100 mg/m^2^ on day 1 followed by fluorouracil 1000 mg/m^2^/d for five days every four weeks) regimens were compared and the median OS was detected better in the DCF arm (9.2 months vs. 8.6 months) [19]. In the same study, rates of febrile neutropenia, ≥ grade 3 leukopenia, and neutropenia were significantly higher in the DCF group [19]. In another phase 3 randomized study in metastatic GC patients, the effects of adding docetaxel to S-1 and cisplatin were investigated and no difference was detected between treatment outcomes [16]. In the same study, grade 3 and higher toxicity were found to be higher in the triple combination (59% vs. 32%).

In the literature, there are various studies comparing DCF and mDCF in first-line treatment in metastatic GC patients [20]. In another study comparing DCF + granulocyte colony-stimulating factor (G-CSF) and mDCF (fluorouracil 2,000 mg/m^2^ intravenously [IV] over 48 hours, docetaxel 40 mg/m^2^ IV on day 1, cisplatin 40 mg/m^2^ IV on day 3, every two weeks), while the median OS was better in the mDCF group (18.8 v 12.6 months; P=0.007), the median PFS was numerically better in the mDCF group, but could not reach statistical significance (mDCF, 9.7 v DCF, 6.5 months) [21]. In the same study, while grade 3-4 toxicity was observed at a rate of 90%, neutropenia with a rate of 45%, and febrile neutropenia in 16% in the DCF arm; grade 3-4 toxicity was 76%, neutropenia was 56%, and febrile neutropenia was 9% in the mDCF arm [21].

There are limited studies comparing FOLFOX and DCF/mDCF regimens in metastatic GC. In the study of Pourghasemian et al., mDCF and FOLFOX-4 were compared in advanced stage gastric adenocarcinoma patients, and no difference was found between the group in terms of the objective response rate (46.98% vs. 35.1%, respectively), OS (13.50 ± 5.94 months vs. 12.61 ± 4.05, respectively) [22]. In addition, while hematological side effects such as neutropenia, neutropenic fever, thrombocytopenia were more common in the mDCF arm, neuropathy was observed more frequently in the FOLFOX-4 arm [22]. Al-Batran et al. [23] compared FLO (oxaliplatin 85 mg/m^2^ and leucovorin 200 mg/m^2^, each as a two-hour intravenous infusion, followed by FU 2600 mg/m^2^ as a 24-hour continuous infusion every two weeks) and FLP (cisplatin 50 mg/m^2^ as a two-hour infusion every two weeks combined with leucovorin 200 mg/m^2^ as a two-hour infusion and FU 2000 mg/m^2^ as a 24-hour infusion every week for six weeks followed by a two-week rest) treatment regimens in their study and they found no difference between the FLO and FLP groups in terms of treatment response rates (25% vs. 35%, respectively), OS (8.8 vs. 10.7 months, respectively), and PFS (6.0 vs. 3.1 months, respectively). In this study, treatment responses between the two groups were examined in patients who received mDCF and mFOLFOX-6 as metastatic first-line therapy, and no difference was found in terms of treatment responses, median OS, and median PFS. Additionally, although dose reduction, delay of treatment, and neutropenic fever were numerically higher in the mDCF arm in our study, they did not reach statistical significance. Considering the above-mentioned studies comparing DCF and mDCF, it can be concluded that mDCF is non-inferior in terms of OS and PFS and its side effect profile is better than DCF.

In our study, the presence of peritoneal metastasis was evaluated as a significant prognostic factor in univariate analysis in terms of OS, but no statistical significance was reached in multivariate analysis. Similarly, in various studies, the presence of peritoneal metastasis in patients has been defined as a poor prognostic factor [24,25]. While there were differences between treatment arms regarding metastasis status (de novo vs recurrent), metastasis status has not been found to be a prognostic factor for OS both in univariate and multivariate analysis. In addition, age, sex, ECOG-PS, histological subtype, presence of liver metastasis, de novo, or recurrent metastasis, number of metastases were not included among prognostic factors associated with OS in this study.

The major limitations of the present study were that its retrospective nature and the relatively small number of patients. In addition, while evaluating the side effect profiles of the patients, apart from neutropenic fever, treatment delay and dose reduction, non-hematological side effects such as neuropathy, nausea, and vomiting could not be evaluated.

## Conclusions

The present study demonstrated that proper patient selection for metastatic GC may give rise to comparable survival rates without increased toxicity. According to our data, the presence of comorbid disease was the major determining factor for the choice of the treatment regimen. Fluoropyrimidine and platinum-based combination therapies are the most commonly used regimens in the first-line treatment of metastatic GC. In addition, docetaxel can be added to the treatment in patients with a high tumor burden and relatively better performance status. In addition, although different modified doses were used in the studies, it could be shown that mDCF is noninferior to DCF. Finally, mFOLFOX-6 and mDCF had similar response rates, OS, PFS, and side effect profiles. Studies with larger numbers of patients and larger populations are needed.
